# A Pilot Interprofessional Education Curriculum for Optimizing Mental Health in Chronic Pain Treatment and Understanding Interprofessional Practice

**DOI:** 10.7759/cureus.67292

**Published:** 2024-08-20

**Authors:** Juliette Perzhinsky, Kathleen A Schachman, Chin-I Cheng, Sally Nagia, Bernard Noveloso, Tamara Sawyer, Brenda L Lepisto, Javeed Sukhera, Elizabeth N Cleek, Margaret S Chisolm

**Affiliations:** 1 Foundational Sciences, Central Michigan University College of Medicine, Saginaw, USA; 2 Nursing, Saginaw Valley State University, Saginaw, USA; 3 Statistics, Actuarial, and Data Science, Central Michigan University, Mount Pleasant, USA; 4 Anesthesiology, Cleveland Clinic Foundation, Cleveland, USA; 5 Family Medicine, Central Michigan University College of Medicine, Saginaw, USA; 6 Knowledge Services, Central Michigan University College of Medicine, Saginaw, USA; 7 Family Medicine, McLaren Greater Lansing, Michigan State University, College of Osteopathic Medicine, East Lansing, USA; 8 Psychiatry, Hartford Hospital, Hartford, USA; 9 Operations, The Arnold P. Gold Foundation, Fort Lee, USA; 10 Psychiatry and Behavioral Sciences, The Johns Hopkins University School of Medicineniversity School of Medicine, Baltimore, USA

**Keywords:** graduate medical education (gme), interprofessional education, opioid use disorder, mental health, chronic pain

## Abstract

Introduction: With the urgent need for clinicians capable of responding to the opioid crisis, an interprofessional education (IPE) pilot curriculum was launched to assess trainee self-efficacy in managing chronic pain and mental health conditions, and attitudes toward interprofessional practice among resident physicians, family nurse practitioners (FNP), and physician assistant (PA) students.

Methods: This study involved the implementation of a pilot curriculum consisting of five interactive IPE sessions. All invited trainees across two academic institutions were asked to complete the assessments. Self-efficacy in managing chronic pain and mental health was measured at baseline and following IPE training using a researcher-developed tool, while attitudes toward interprofessional practice were measured with the Attitudes Toward Health Care Teams scale. Resident physicians were compared to FNP/PA students to examine differences between groups and within groups over time.

Results: The final analysis involved 25 trainees who attended at least one IPE training session and completed pre-session and post-session surveys. The total pre-session survey and post-session survey response rate was 37.5% (n=36). Self-efficacy in chronic pain management improved among the resident physician (mean=3.85 ±0.40) and FNP/PA groups (mean=3.84±0.46) (p=0.05 and p=0.001), respectively. Self-efficacy in mental health management was not significantly improved among resident physicians (mean=3.41±0.49, p=0.48), but improved among FNP/PA students (mean=3.46±0.31, p<0.001). There was no difference in attitudes toward interprofessional practice.

Conclusion: While IPE training did not result in attitudinal changes toward interprofessional practice, it shows potential for improving self-efficacy in managing chronic pain and mental health, particularly among FNP/PA trainees. This study was limited by a small sample size of trainees included in the final analysis.

## Introduction

The opioid epidemic began in the late 1990s when fatalities linked to opioid pain medications increased at an alarming rate with increased prescribing of opioids by clinicians as a major contributing factor [1,2]. Studies have shown that patients with mental health conditions suffered disproportionate morbidity and premature death and that untreated severe mental illness correlated with rising opioid-related deaths [3]. Furthermore, the declaration of the coronavirus disease 2019 (COVID-19) public health emergency resulted in a rapid increase in opioid-related mortality with over 100,000 deaths in 2020 [4]. As millions of Americans continue to suffer from chronic pain and comorbid mental health conditions placing patients at risk of opioid use disorder (OUD) [5], it is imperative for graduate medical education (GME) and other health professions programs to train interprofessional healthcare teams in safe opioid prescribing practices and the timely recognition of addiction [6].

GME regarding chronic pain management and OUD offers a unique opportunity to influence a practitioner’s lifetime approach to the care of patients with these conditions. However, the emphasis on required or standardized education regarding opioid prescribing has been highly variable among clinical training programs [7]. Many clinicians lack the skills needed to screen, assess, treat, and refer patients with signs of opioid misuse [8]. Primary care clinicians, such as family medicine physicians, physician assistants (PAs), and nurse practitioners (NPs) may be the first healthcare professionals to come in contact with a patient with chronic pain struggling with opioid misuse versus OUD [9]. Unfortunately, most clinicians do not have significant training in chronic pain management or addiction medicine [10]. A 2017 study surveying 227 United States (US) family medicine residency training program directors showed that only one-third of these programs had addiction medicine education integrated into their curriculum; furthermore, program directors ranked the implementation of addiction medicine curricula into their program as only a moderate priority [11]. PA programs demonstrate an even wider variation of training in opioid education. In a 2019 study, 49 (55.7%) of 88 PA educators who responded to a survey across the US indicated that their PA programs had a mandatory opioid education component; when such curricula were included, they were typically single didactic sessions of one to three hours [12].

Some clinical training programs have recognized the need for more educational opportunities regarding chronic pain and safe opioid prescribing and piloted curricula to help mitigate these educational gaps. In a 2017 study, internal medicine residents reported improved self-confidence in communicating with and managing patients with chronic pain and opioid misuse after undergoing two educational sessions [13]. Similarly, another program instituted a week-long curriculum for internal medicine residents on prescribing medication for OUD; qualitatively, these residents felt this specialized curriculum helped them gain a better understanding of OUD and increased their confidence in treating patients with this condition [14]. Despite these advances, gaps still exist in medical training programs [15].

Another approach is the use of integrated interprofessional education (IPE) models throughout different education programs to leverage training to enhance teamwork and collaboration across trainee disciplines [16]. IPE is defined as two or more healthcare professionals learning together with the goal of improving collaboration amongst healthcare teams and, ultimately, improving patient care [17]. Applying IPE to address the complex issues of chronic pain, comorbid mental health conditions, and OUD across training programs may be an evolving strategy if deemed feasible and replicable. IPE has the potential to provide a novel approach to opioid curricula. For example, a 2016 article by Maranzan et al. highlights the importance of integrating IPE training as a means for reducing mental illness stigma amongst healthcare professionals [18]. Furthermore, a 2013 study recruited 151 healthcare professionals working in an arthritis clinic to undergo an interprofessional training program; results from participants’ self-assessments showed statistically significant increases in survey scores in knowledge, skills, and healthcare team collaboration in chronic disease settings [19]. Thus, chronic pain, mental illness, and OUD are patient conditions that may be addressed best using a multidisciplinary (and interprofessional) approach as they involve complex health-related and social needs [20].

We have entered an era in health care where the deliberate practice of providing compassionate care is a mitigation strategy for addressing opioid addiction, especially in circumstances when patients received legitimate treatment for chronic pain with opioid therapy preceding the development of OUD [21]. The COVID-19 pandemic demonstrated the urgency of improving the care of vulnerable patients with addiction [22]. Even though this pilot curriculum preceded the occurrence of COVID-19, its context is still relevant. Health profession educators are obligated to imprint the humanistic delivery of care into future trainees who will inevitably become independent physicians and health care practitioners. This approach aligns with the patient-centered care principles set forth by the Picker Institute Always Events® [23], specifically for educating trainees on enhancing communication involving patients with chronic pain and mental health co-morbidity, who are at the highest risk of having opioid misuse or OUD, and ensuring effective coordination and integration of care to optimize health outcomes.

This project aimed to pilot a curriculum to address chronic pain and the opioid crisis while expanding the curriculum to interprofessional trainees. We hypothesize that participation in interdisciplinary sessions would result in higher levels of trainee self-efficacy in managing chronic pain and comorbid mental health conditions, interprofessional team-based practice, and less emphasis on a physician-centric management approach. The central theme of the IPE curriculum was to build trust, increase learner self-efficacy, and foster patient-centered interprofessional collaboration when treating vulnerable patients consistent with core interprofessional competencies as defined by the Interprofessional Education Collaborative Expert Panel [24].

The objectives of the study included: (i) to evaluate learner self-efficacy with managing chronic pain and mental health conditions (including the screening and recognition of OUD and other substance use disorders), and (ii) to assess attitudes on interprofessional practice as part of the pilot IPE curriculum. A convenience sample of interprofessional trainees who attended at least one IPE session and completed both the pre-session and post-session surveys was included in the final analysis.

This curricular project was presented as a workshop at the Accreditation Council for Graduate Education Annual Educational Conference in Orlando, Florida, United States, on March 9, 2019, in memory of Thomas Martin Slomka (1979-2019).

## Materials and methods

We developed and implemented a pilot IPE curriculum to train primary care resident physicians, family nurse practitioner (FNP) students, and PA students to deliver empathic care and to maintain a therapeutic alliance with patients with chronic pain and mental health co-morbidities, patient groups who are both at high risk of OUD. The Office of Research Compliance, Central Michigan University acknowledged and exempted the study from approval (submission: 923801-2). All participants gave written consent (Appendix A).

Clinical trainees from four primary care-oriented residency programs, an FNP program, and a PA program, located at Central Michigan University College of Medicine (residency training programs and PA program) and Saginaw Valley State University (FNP program), both campuses in Saginaw, Michigan, were invited to participate in this pilot IPE curriculum. The pilot was delivered over a six-month period from December 2017 to May 2018, offering five IPE training sessions spanning 2.5-3 hours in duration with session-specific objectives addressing opioid crisis-relevant content (Table 1).

**Table 1:** Pilot IPE curriculum: five sessions with respective educational learning objectives. IPE: inteprofessional education

Session Objectives
Session 1: Communication & Motivational Interviewing Strategies to Address Chronic Pain
1) Practice a systematic approach of obtaining a pain history in a patient with chronic pain and concurrent mental illness. 2) Apply motivational interviewing techniques to help promote patients’ self-management skills in dealing with chronic pain beyond just taking pain medication. 3) Identify and address openly with patients any aberrant behaviors, discrepant urine drug screens/prescription monitoring database (PMDB) reports/pill counts, or pain agreement violations.
Session 2: Applying Patient Safety Methods for Safely Prescribing Opioids
1) Review statistics, physiology and risks associated with opioid addiction. 2) Define specific patient safety methods in safe opioid prescribing. 3) Apply Team Strategies and Tools to Enhance Performance and Patient Safety (Team STEPPS) for collaborative practice when prescribing opioids.
Session 3: Understanding the Nature of Addiction
1) Name the four “perspectives” of psychiatry. 2) Describe the conceptual triad associated with each perspective. 3) Apply all four perspectives to the formulation of a patient presenting with substance use.
Session 4: Screening Brief Intervention and Referral to Treatment (SBIRT)
1) Understand SBIRT's role in identifying individuals who do not meet the criteria for a substance use disorder. 2) Understand the role of SBIRT as a general screen for all patients regardless of an identified disorder. 3) Apply the concepts of SBIRT in a variety of clinical settings.
Session 5: Biopsychosocial Model & Impact Bias in Opioid Treatment
1) Review the biopsychosocial model for the management of chronic pain. 2) Explore implicit social cognition influences of health professional attitudes and behaviors towards individuals who use opioids. 3) Discuss strategies for healthcare professionals to recognize and manage implicit biases towards individuals who use opioids.

Sample selection and participants

We sent all invited trainees a pre- and post-session survey. A total of 155 trainees from four primary care-based residencies (Family Medicine, Internal Medicine, Obstetrics & Gynecology, and Psychiatry), an FNP program, and a PA program were invited to the IPE sessions. The FNP program required attendance at at least three of the five sessions or, alternatively, the option to write a paper on chronic pain. Trainees who were rotating on core inpatient rotations did not attend the sessions due to their inherent clinical/educational commitments. Geographic constraints existed on travel to on-site training, especially for FNP/PA students who clinically rotated across different regions in the state.

**Figure 1:**
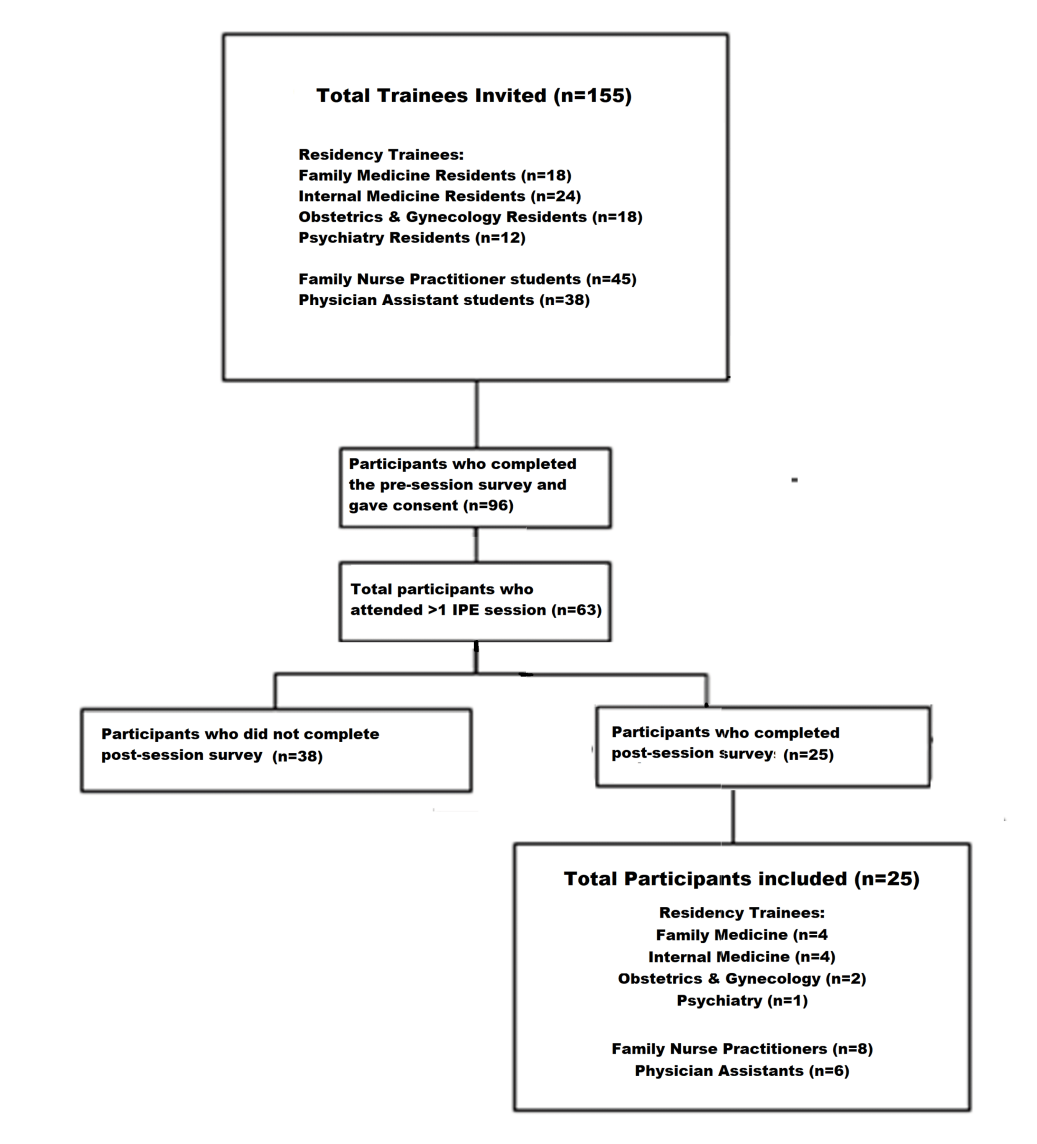
Flowchart showing the selection process of the study

The participants were divided into two groups: (i) residents and (ii) FNP/PA students. Study inclusion was limited to trainees across all six training programs who completed both the pre and post-session survey assessments and participated in at least one IPE session. Of the 155 trainees invited to participate, 96 (61.9%) responded to the baseline (pre-session) survey. Of these, a total of 63 participants attended at least one IPE session. Attendance ranged from 21 to 38 participants at each of the five in-classroom IPE training sessions. Of the 63 trainees who attended at least one IPE session (having earlier completed the pre-session survey), 25 completed the post-session survey, comprising 11 residents, eight FNP students, and six PA students. The process is shown in Figure 1. Thus, a total of 25 trainees who completed both the pre-session and post-session surveys and also attended at least one IPE session were included in the final analysis.

Data collection

We administered a 40-item pre-session and post-session survey prior to the IPE sessions and after the IPE sessions, respectively. There were two additional items in the post-session survey regarding whether they participated in any of the IPE sessions and how many sessions they attended.

The 40-item survey consisted of the 20-item Attitudes Toward Health Care Teams (ATHCT) survey and a novel 20-item survey developed and piloted to assess the trainees’ level of confidence in managing mental health and chronic pain (See Appendix B).

The ATHCT is a validated assessment scale that focuses on the domains of addressing team quality of care and team processes consisting of 14 items, along with six items on physician centrality [25].

With regard to the novel 20-item self-efficacy survey, we conducted the pilot six months prior to the pilot study, which established a Cronbach alpha of 0.90, consistent with good internal consistency. Self-efficacy was measured by participants’ responses on a five-point Likert scale (1-5) to 10 items relating to their “level of confidence in managing mental health conditions” and 10 questions relating to their “level of confidence related to chronic pain.”

The combined 40-item survey was anonymous and utilized a self-reported (de-identified) participant code for pre-session and post-session comparison.

Post-IPE session evaluation

Apart from the pre-session and post-session surveys, all participants of all IPE sessions were also asked to fill out an evaluation feedback questionnaire (Appendix C). Not all the participants responded to the post-course evaluation questions from each of the IPE sessions. Please note that the evaluation feedback was different from the post-session survey. Although attendance at each of the five IPE training sessions varied, the final data analysis only included the participants who completed both the pre- and post-session surveys (40-item survey), which is a different instrument, as described earlier.

Statistical analysis

Statistical analysis was conducted using IBM SPSS Statistics for Windows, Version 26.0 (Released 2019; IBM Corp., Armonk, New York, United States) [26]. Descriptive statistics included mean±standard deviation (SD) for variables measuring the level of confidence and attitudes and percentage (counts) for the proportion of trainees from various training programs in the study. We used the two-sample t-test to compare the average score of level of confidence and attitudes between primary care residents and FNP/PA students. The alternative Welch’s t-test was used when the assumption of equality of variance was violated. WA paired t-test was adopted to examine whether IPE training changed the average score of level of confidence and attitudes. One-way repeated ANOVA model measured the interaction effect between pre-/post-IPE sessions and the training program. Significance was achieved with the analytical results with p-values less than or equal to 0.05.

## Results

IPE session evaluations

Participation ranged from 21 to 38 participants at each of the five in-classroom IPE training sessions (an average of 30 participants per IPE training session), with the majority of sessions occurring during the second semester of the academic year. After each IPE session, participants were asked to submit their evaluation feedback to evaluate the effectiveness of the individual learning sessions. Across all five IPE sessions, an average of 92% of respondents (n=29) recommended the course to their colleague, 93% of the respondents (n=28) felt that IPE should be a part of all healthcare training programs, and 94% (n=28) of the respondents felt that the IPE session enhanced their skills of working with other disciplines.

IPE survey analysis of self-efficacy: managing mental health and chronic pain

Table 2 compares the results of the pre-session and post-session self-efficacy questionnaire. The post-session survey self-efficacy assessment assessing the level of confidence in managing mental health conditions did not show a significant difference in resident physicians (mean=3.41±0.49, p=0.48), but there was a statistically significant difference with the FNP/PA students (mean=3.46±0.31, p=<0.001) and between both groups in the pre-session and post-session survey analysis (p=0.003). The post-session survey showed an improvement in trainee self-efficacy with managing mental health conditions for the FNP/PA students as compared to the residents.

**Table 2:** Comparison of pre-session and post-session self-efficiency scores of confidence in managing mental health and chronic pain SOAPP: Screener and Opioid Assessment for Patients with Pain; ORT: Opioid Risk Tool

Level of confidence		Pre-session composite score, mean±SD	Post composite score, mean±SD	p-value
Level of confidence in managing Mental Health				
Q1	In my ability to manage mental health conditions	3.28±0.89	3.72±0.46	0.02
Q2	That I have sufficient skills in diagnosing mental health conditions	3.24±0.88	3.76±0.52	0.03
Q3	That my training program is preparing me well to accurately diagnose and treat mental health	3.36±0.95	3.72±0.68	0.05
Q4	In my ability to use the Patient Health Questionnaire (PHQ-9) in my clinical practice setting to assess for depression	4.04±0.89	4.52±0.51	0.01
Q5	In my ability to use the Generalized Anxiety Disorder 7-item (GAD-7) questionnaire in my clinical practice setting to assess for anxiety	3.72±0.98	4.48±0.65	0.001
Q6	In my ability to use the Mood Disorder Questionnaire (MDQ) in my clinical practice setting to assess for a mood disorder	3.24±0.88	3.76±0.93	0.02
Q7	In my ability to effectively treat a full spectrum of mental health conditions	3.00±1.04	3.20±0.87	0.46
Q8	With providing patients timely access to psychiatric care when I refer them	3.32±1.07	3.20±0.96	0.56
Q9	That mental illness in our society is no longer stigmatized	2.28±0.89	1.80±0.76	0.02
Q10	That patients with mental illness receive equitable care	2.76±1.05	2.20±0.96	0.03
Level of confidence in managing Chronic Pain				
Q11	I have sufficient training to effectively manage chronic pain	2.92±0.76	3.52±0.65	0.001
Q12	In my ability to discuss buprenorphine or naloxone treatment to patients with opioid use disorder	2.68±1.03	3.20±0.96	0.02
Q13	In my ability to discuss behavioral therapy to patients who have chronic pain	3.32±0.90	3.92±0.57	0.02
Q14	In my ability to discuss and prescribe a home naloxone kit when high-dose opioids are used to treat chronic pain	2.32±1.07	3.04±0.98	0.01
Q15	In my ability of when to recommend pain specialty referral when patients have chronic pain	3.40±0.96	4.20±0.71	0.002
Q16	In my ability to utilize a state prescription system to verify opioid usage (even if checked by a team member) when prescribing opioids	3.80±1.15	4.32±0.56	0.03
Q17	In my ability to use an opioid risk assessment tool (ie, SOAPP, ORT) prior to prescribing opioids	2.84±1.25	3.88±0.78	<0.001
Q18	In my ability to explain to patients the risks of using marijuana and opioids together to treat pain	3.32±1.03	3.88±0.88	0.008
Q19	In my ability to explain to patients the risks of using opioids and benzodiazepines together	3.72±0.89	4.40±0.50	0.005
Q20	In my ability to screen patients for substance abuse using the Screening, Brief Intervention & Referral to Treatment (SBIRT) tool	2.92±1.04	4.08±0.70	<0.001

IPE survey analysis of ATHCT survey of interprofessional practice: team quality of care and team process and physician centrality

The first part of the ATHCT survey on the interprofessional practice domain consisted of a 14-item survey. Neither the resident nor FNP/PA student cohorts demonstrated any change in improving team quality of care or processes. We identified no statistical significance between and among the residents and FNP/PA students in the pre-session and post-session surveys (p-values 0.82 and 0.10, respectively) on the interprofessional practice of improving team quality of care.

Similarly, the second part of the ATHCT survey consisted of the six-question physician centrality domain. We found no difference in attitudes toward physician centrality before and after attendance between and among the residents and FNP/PA students with the pre-session and post-session assessment (p-values 0.38 and 056, respectively). We found no change in interprofessional practice attitudes when we compared the resident and FNP/PA student cohorts, although a core focus of this pilot IPE curriculum was to potentially enhance the use of interprofessional teamwork and communication. 

Pre-IPE and post-IPE sessions combined scores

Table 3 provides a summative review of the pre-session and post-session survey results among the study group, and additionally explores the survey domains that showed a statistical difference between the cohorts, and the areas in which there was no difference in trainee self-efficacy.

**Table 3:** Pre- and post-IPE session comparison of mean scores in managing mental health and chronic pain combined with ATHCT survey assessment mean scores between primary care residents and FNP/PA students. ^*^comparison between pre-IPE and post-IPE based on paired t-test; ^ǂ^comparison between primary care residents and FNP/PA students based on two sample t-test; ^¥^ interaction effect between training program and self-efficacy/interprofessional practice based on one-way repeated ANOVA. Primary Care Residents (n=11) include four from Family Medicine, four from Internal Medicine, two from Obstetrics & Gynecology, and one from Psychiatry. Family Nurse Practitioner (FNP)/Physician Assistant (PA) students (n=14) include eight FNP and six PA students, respectively. ATHCT: Attitudes Toward Health Care Teams; IPE: interprofessional education

		Self-Efficacy						Interprofessional Practice					
Training Program	n	Mental Health composite score, mean±SD			Chronic Pain composite score, mean±SD			ATHCT Team Quality of Care & Processes score, mean±SD			ATHCT Physician Centrality score, mean±SD		
		Pre-IPE	Post-IPE	p-value*	Pre-IPE	Post-IPE	p-value*	Pre-IPE	Post-IPE	p-value*	Pre-IPE	Post-IPE	p-value*
Primary Care Residents	11	3.54±0.61	3.41±0.49	0.48*	3.47±0.51	3.85±0.40	0.05*	3.73± 0.31	3.77±0.517	0.82*	3.58±0.57	3.46±0.30	0.38*
FNP/PA students	14	2.98±0.43	3.46±0.31	<0.001*	2.85±0.72	3.84±0.46	0.001*	3.56±0.31	3.71±0.29	0.10*	2.99±0.31	3.08±0.57	0.56*
p-valueǂ		0.01ǂ	0.77ǂ	0.003¥	0.02ǂ	0.99ǂ	0.05¥	0.17ǂ	0.75ǂ	0.46¥	0.004ǂ	0.06ǂ	0.31¥

Regarding self-efficacy in managing chronic pain and safely prescribing opioids, we found statistically significant differences in pre-session and post-session scores for the residents (mean=3.85±0.40, p=0.05) and FNP/PA students (mean=3.84±0.46, p=0.001). The results suggest a greater improvement in self-efficacy in chronic pain management observed among FNP/PA students compared to the resident physicians (p=0.05). Whereas, resident physicians had a higher level of baseline self-efficacy in chronic pain management (mean = 3.47±0.51) as compared to FNP/PA students (mean=2.85 ± 0.72).

**Figure 2:**
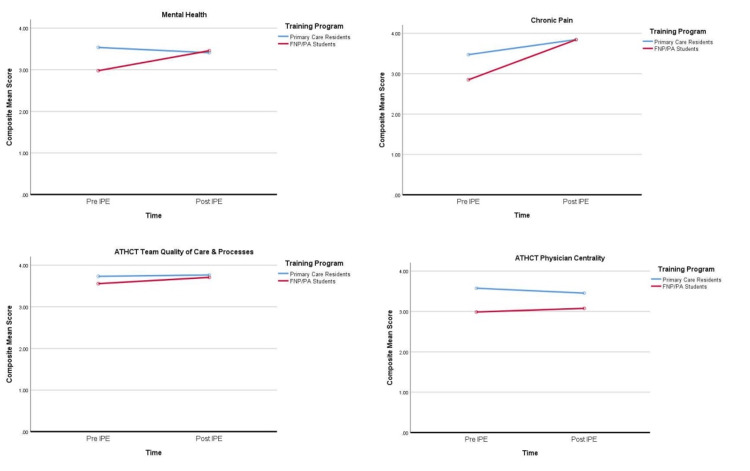
Comparison graphs of composite mean scores of self-efficacy and ATHCT domains for primary care residents and FNP/PA trainees. FNP: Family Nurse Practitioner; PA: Physician Assistant; ATHCT: Attitudes Toward Health Care Teams

Figure 2 graphically represents the pre-session and post-session scores in all domains of the survey where both ATHCT interprofessional practice domains are shown as horizontal lines for both groups of trainees.

## Discussion

Our pilot IPE curriculum sought to train resident physicians, FNP, and PA students in a patient-centered approach to the assessment of chronic pain, the recognition of mental health conditions including OUD, and the safe prescribing of opioids. We hypothesized that attendance at one or more of the five interdisciplinary sessions offered over the course of an academic year would translate into higher levels of trainee self-efficacy in managing chronic pain and possible comorbid mental health conditions. An additional aim was that the pilot IPE curriculum would shift trainee attitudes with more emphasis on interprofessional team-based practice and less emphasis on a physician-centric management approach.

We found that trainees who attended a least one session reported higher self-efficacy in managing chronic pain after session attendance compared to before session attendance. Specifically, session attendees felt more comfortable screening patients using an opioid risk assessment tool, employing the (Screening-Behavioral Intervention-Referral to Treatment (SBIRT) instrument, and explaining the risks of marijuana and opioids to treat pain. The statistical finding showed that the IPE session(s) had an impact on improving learner confidence in chronic pain management (Table 3). When comparing the residents to the FNP/PA students, both groups achieved statistical significance indicating that the IPE sessions improved learner self-efficacy in managing chronic pain regardless of the training program. This may lend to the fact that the core educational content of the IPE training sessions was predominantly focused on addressing the management of chronic pain.

As for assessing a change in the level of confidence in managing mental health conditions, only the FNP/PA students, but not the resident physicians, demonstrated an improvement in self-efficacy in managing mental health conditions (Table 3). It is possible to conclude that resident trainees who participated in the IPE sessions already were cognizant of the management of mental health conditions and co-morbidity through prior medical school training. Since this pilot curricular project was aimed at primarily focusing on training in the management of chronic pain and safe opioid prescribing, another possible explanation for the finding is that the resident physicians were already taught the general management of mental health conditions elsewhere in their respective curricula.

Our results are unexpected regarding attitudes toward working in a healthcare team. Participation in an IPE session did not yield any difference in attitudes in interprofessional practice, even though participants acknowledged improvement in patient care quality from an interprofessional approach in the individual session evaluations. These unanticipated findings lend pause for self-reflection on refining the content of our pilot curriculum to convey that all members of an interprofessional team share responsibility for quality patient care and that such interprofessional care should not be overly time-consuming. Feedback received on the session evaluations mentioned that trainees being assigned to consistent interprofessional groups could have been beneficial; however, this was difficult to coordinate with the lack of consistency with trainee attendance at the IPE sessions as discussed.

Our pilot study of a curriculum addressing chronic pain and opioid use harnessed the expertise of internal faculty and external guest speakers who facilitated five in-person, interprofessional sessions for resident physicians, FNP, and PA students. Our educational approach differed from another pilot study in which resident physicians of a single internal medicine residency program, over six one-hour required educational sessions, were tasked with reviewing and presenting four journal articles relating to opioid prescribing for chronic pain, in addition to discussing their new clinic protocol for treating patients with chronic non-cancer pain [27]. Whereas Vettese et al.'s study involving internal medicine residents specifically assessed medical knowledge and motivation to change behavior regarding chronic pain management and opioid prescribing following a single three-hour educational workshop [28], we focused on trainee self-efficacy without objectively assessing medical knowledge. Several of our sessions involved a didactic component followed by breakout discussions in assigned small groups, with care to include resident physicians, FNP, and PA students in each small group. Another studied educational approach involving internal medicine residents entailed standardized patient and faculty feedback on an objective structured clinical exam (OSCE) immediately following a didactic lecture, with significant improvement in residents’ reported confidence in safe opioid prescribing [29].

Limitations

The successful implementation of our pilot IPE curriculum within the grant funding schedule required a considerable amount of expedited coordination among the schedules of four residency programs, a PA training program, and an FNP training program at another institution, not to mention the coordination of guest speakers. There were varying levels of commitment among the training programs to encourage attendance among trainees who have other competing clinical training demands. Whereas the FNP students were required to attend at least three of the five sessions, the IPE sessions were not approved as a core curricular component of any of the GME residency training programs or the PA program. Participating resident physicians and PA students, for instance, were essentially a convenience sample of trainees on flexible ambulatory rotations. Although there was some overlap in the material of the IPE sessions, the sessions by no means were intended to be interchangeable. Trainees exposed to our material at the beginning of the academic year very well could have different perceptions of self-efficacy compared to trainees who attended a session later in the academic year after having more clinical experience. Moreover, trainees who attended more sessions likely would differ in self-efficacy from trainees who attended just one session. Our small sample size limits our ability for meaningful comparison of trainee responses based on training program due to challenges with engaging a consistent cohort of participants. 

## Conclusions

Although this IPE training curriculum to address chronic pain management and optimizing mental health did not demonstrate a change in attitudes for trainees toward interprofessional practice, it shows potential for improving learner self-efficacy in managing chronic pain and mental health. Our goal was that this pilot study should further advocate for education on chronic pain management, mental health co-morbidity, and OUD in vulnerable patients as part of a required curriculum for resident physicians and health professions students. Although this pilot IPE curriculum faced several logistical challenges, advanced planning and coordination as part of a core curriculum may yield enhanced efficacy and a change in attitudes toward interprofessional teamwork and practice. Feedback from the post-session evaluations gravitated towards hearing more personal testimonials from individuals who had experience with chronic pain or addiction. Additionally, providing pre-recorded and virtual interprofessional discussions of the material may widen learner access to key educational content for trainees who will ultimately inherit the care of high-risk patients during the opioid crisis.

## Figures and Tables

**Figure 3:**
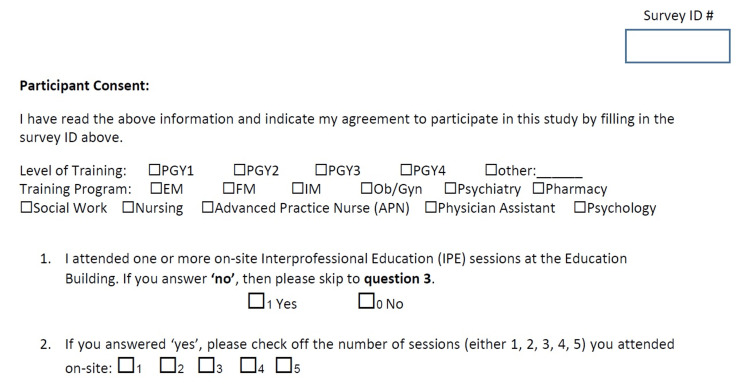
Questionnaire: Mental Health, Chronic Pain & Interprofessional Care This survey is being administered to assess your confidence in managing mental health conditions and chronic pain in any clinical setting and your level of agreement with interprofessional practice. It has been waived by the Institutional Review Board (IRB) and responses to this survey are strictly voluntary. This information may guide future curriculum changes. Please answer the following questions honestly. All survey information is anonymous and will remain anonymously analyzed. All information gathered in this study will be kept confidential and there will be no reference to any individual identifiers linking you to this survey. The survey should take approximately 10 minutes to complete. Please provide the following information in the above upper right-hand corner box on each page (Survey ID): (1) the first letter of your mother's maiden name, (2) number of the month of your birthday (as 2 digit month), and (3) the first 2 numbers of your family’s home address upon entering residency. Thank you for your participation.

**Figure 4:**
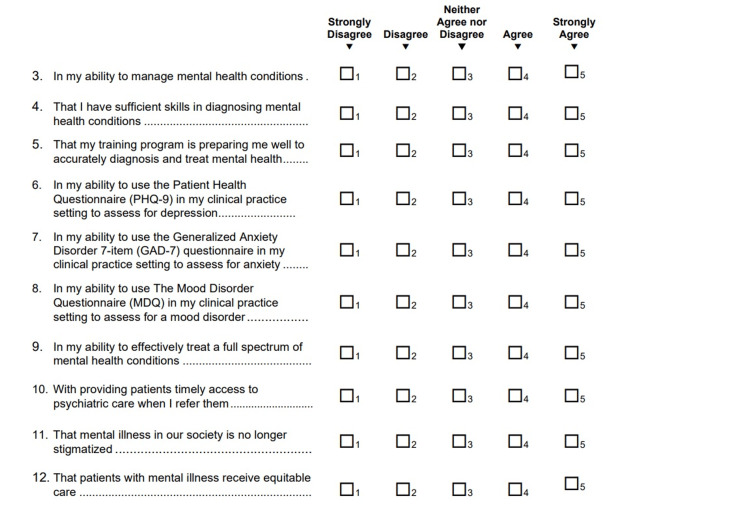
Please answer the following statements with your level of confidence related to chronic pain. If an item is not applicable to you, please leave the item blank. Check one box for each statement: I AM CONFIDENT…

**Figure 5:**
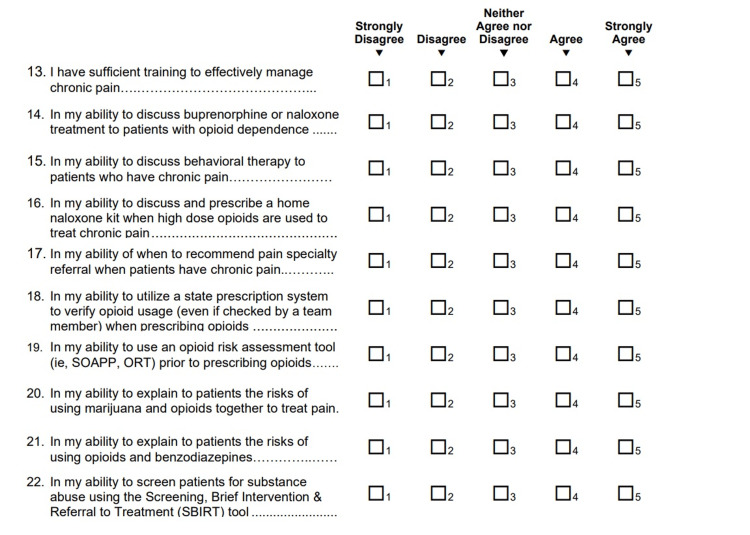
Please answer the following statements with your level of confidence in managing mental health conditions. If an item is not applicable to you, please leave the item blank. Check one box for each statement: I AM CONFIDENT…

**Figure 6:**
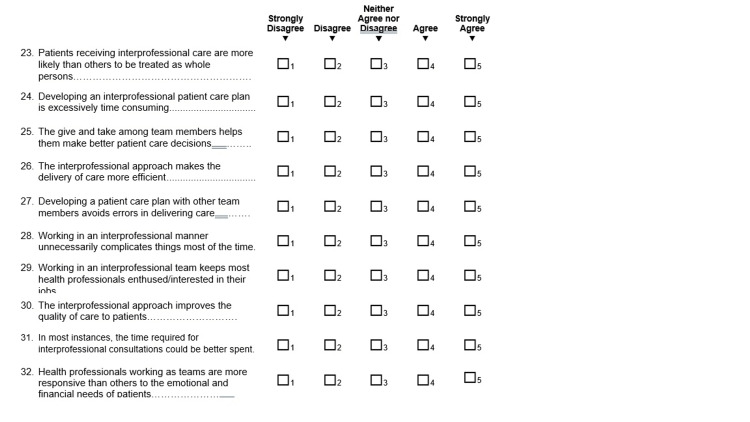
Please answer the following statements with your level of agreement related to interprofessional practice. If an item is not applicable to you, please leave the item blank. Check one box for each statement.

**Figure 7:**
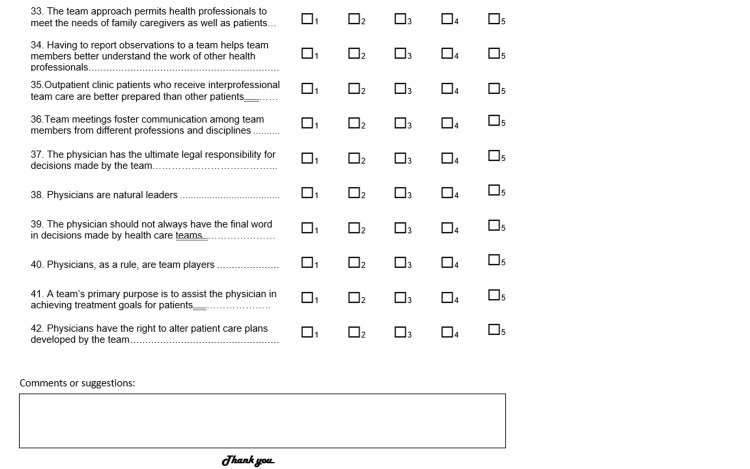
Please answer the following statements with your level of agreement related to interprofessional practice. If an item is not applicable to you, please leave the item blank. Check one box for each statement.

**Figure 8:**
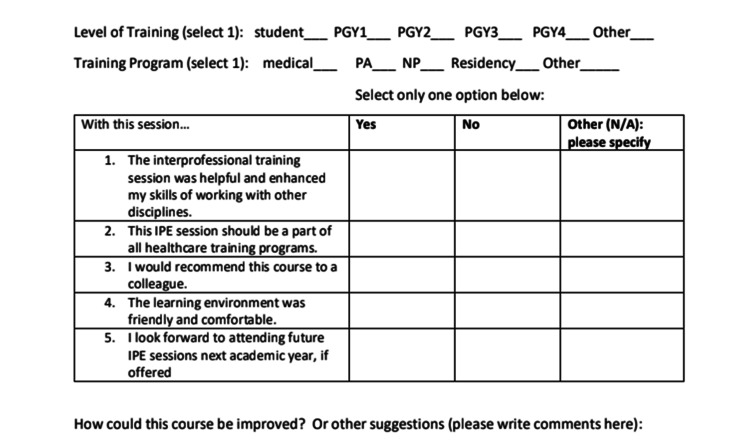
Evaluation/Feedback Form

## Appendices

Appendix A: survey description and survey consent

This is the combined researcher-developed survey consisting of 20 items on assessing self-efficacy in mental health and chronic pain management, opioid prescribing, and the Attitudes Toward Health Care Teams 20-item survey to assess interprofessional practice. The post-session survey had two additional questions on whether the trainee attended any of the IPE training sessions and the number of sessions attended.

Appendix B: 40-item questionnaire

The 4-part survey for assessing the level of confidence in managing mental health conditions and chronic pain (20 items) and the ATCHT survey (20 items).   

Appendix C: post-IPE session evaluation form
